# Incidence, Risk Factors, and Outcomes of Rhabdomyolysis in Hospitalized Patients With COVID-19 Infection

**DOI:** 10.7759/cureus.19802

**Published:** 2021-11-22

**Authors:** Isam Albaba, Amit Chopra, Ali H Al-Tarbsheh, Paul J Feustel, Mohammed Mustafa, Jozef Oweis, Sai Anoosh Parimi, Fabiana M Santelises Robledo, Swati Mehta

**Affiliations:** 1 Internal Medicine, Albany Medical Center, Albany, USA; 2 Department of Pulmonary and Critical Care Medicine, Albany Medical Center, Albany, USA; 3 Department of Research, Albany Medical Center, Albany, USA; 4 Nephrology and Internal Medicine, Albany Medical Center, Albany, USA

**Keywords:** covid-19, : acute kidney injury, severity, incidence, outcome, mortality, physical therapy rehabilitation, rhabdomyolysis

## Abstract

Introduction: There is a paucity of studies examining the prevalence and clinical characteristics of rhabdomyolysis in hospitalized patients with COVID-19 infection. The purpose of this study is to examine the incidence, clinical characteristics, and outcome of hospitalized patients with COVID-19 infection who develop rhabdomyolysis.

Methodology: This is a single-center retrospective analysis of all hospitalized patients with COVID-19 admitted between March 8, 2020, and January 11, 2021. All patients with creatinine kinase (CK) levels available during the hospital admission were included. Rhabdomyolysis was defined as an elevation in CK level higher than five times the upper limit of normal (i.e., 1125 U/L). We compared clinical characteristics and outcomes of patients who developed rhabdomyolysis with patients who did not develop rhabdomyolysis.

Results: The incidence of rhabdomyolysis in hospitalized patients with COVID-19 infection was 9.2%. There was no significant difference noted in comorbidities and clinical characteristics between the two groups. Moreover, there was no significant difference noted in the presence of severe COVID-19 infection (72.7% vs 54.6%, p = 0.1), mortality (27.3% vs 23.9%, p = 0.72), acute kidney injury (59.1% vs 42.7%, p = 0.14), or need for intensive care unit (ICU) care (72.7% vs 51.4%, p = 0.051). However, a higher percentage of patients in the rhabdomyolysis group required physical rehabilitation after discharge (40.9% vs 19.3%, p = 0.02).

Conclusion: The overall incidence of rhabdomyolysis in hospitalized patients with COVID-19 infection was high (9.2%). The presence of rhabdomyolysis was not associated with the increased severity of the disease. Patients with rhabdomyolysis more frequently required physical rehabilitation compared to those without rhabdomyolysis.

## Introduction

Coronavirus disease 2019 (COVID-19) is primarily a respiratory illness [1]. However, almost every organ system can be involved. Many studies have focused on respiratory, cardiovascular, renal, neurological, and gastrointestinal system involvement [2-7]. Little is known about the clinical relevance of rhabdomyolysis in COVID-19 [8-12]. Only one retrospective study to date has examined the incidence and outcome of rhabdomyolysis in patients with COVID-19 infection [13]. The study showed an increased incidence of acute kidney injury (AKI) in patients with rhabdomyolysis. However, the need for physical rehabilitation was not assessed. It is possible that COVID-19 infection may result in muscle inflammation and degradation, which may result in muscle weakness requiring the need for physical rehabilitation.

The purpose of this study is to examine the incidence of rhabdomyolysis in hospitalized patients with COVID-19 infection. In addition, we also describe the demographic and clinical characteristics of patients with COVID-19 infection who develop rhabdomyolysis. To conclude, we compare the outcomes of patients with and without rhabdomyolysis.

## Materials and methods

This retrospective observational study was conducted in a tertiary care center in the capital region of New York state. The study was approved by an Institutional Review Board Committee and registered under protocol number 5825. Data search was performed through the hospital’s electronic medical record (EMR) registry. Subjects were selected by applying a filter search for any patient with lab-confirmed COVID-19 during admission.

Patient data collected included (a) demographics: age, sex, ethnicity, and body mass index (BMI); (b) comorbidities: chronic obstructive pulmonary disease, diabetes mellitus, end-stage renal disease, hypertension, coronary artery disease, and cancer; (c) history of smoking; (d) COVID-19-specific treatments received: corticosteroids, remdesivir, and therapeutic anticoagulation; (e) laboratory data: creatine kinase (CK) and creatinine levels; and (f) outcome: in-hospital mortality, requirement for intensive care unit (ICU) care, duration of hospitalization, duration of stay in the ICU, development of AKI, use of dialysis, need for mechanical ventilation, use of vasopressors, and requirement for physical rehabilitation after discharge.

Inclusion criteria were (a) patients hospitalized between March 8, 2020, and January 11, 2021, with a positive severe acute respiratory syndrome coronavirus 2 (SARS-CoV-2) polymerase chain reaction assay of nasopharyngeal swab and (b) at least one inpatient CK level measurement. Any COVID-19 patient without a CK level measurement was excluded from the study.

CK levels are widely used among clinicians for the diagnosis of rhabdomyolysis [14]. For the purpose of our study, rhabdomyolysis was defined as a CK level more than five times the upper limit of normal. The upper limit of normal was set at 225 U/L in the institution’s laboratory. Kidney Disease Improving Global Outcomes (KDIGO) criteria were used to define AKI [15]. Baseline creatinine was determined by the most recent level available within the last seven to 365 days prior to presentation or, if not available, was based on sex and ethnicity using predetermined estimation values by KDIGO [15]. AKI was defined when there was an increase in the serum creatinine level of 0.3 mg/dl over a 48-hour period or a 50% increase in baseline creatinine level. Severe COVID-19 was defined as death, ICU stays, need for mechanical ventilation, or AKI requiring dialysis.

Subjects were divided into two groups based on the presence or absence of rhabdomyolysis. The group without rhabdomyolysis served as the control group. Continuous variables were summarized as mean values and categorical variables as percentages. Demographic data, clinical characteristics, CK levels, and outcome measures were summarized in tables. CK level trends of the rhabdomyolysis group were graphically represented against time. For categorical data, statistical significance was assessed by contingency tables and chi-square analysis or Fisher’s exact test (if expected frequencies were less than 5). For continuous variables, t-tests were used for normally distributed data (age, BMI) and Mann-Whitney test for skewed data (length of stays).

## Results

A total of 996 patients were hospitalized with COVID-19 infection during the analysis period; 240 patients had at least one CK level checked, out of which 22 (9.2%) were found to have rhabdomyolysis.

Table 1 shows the demographic and clinical characteristics of patients with and without rhabdomyolysis. There was no significant difference between the mean ages of the two groups (59.6 vs 64.4, p = 0.21). There were more male patients in the rhabdomyolysis group than the control (81.8% vs 57.3%, p = 0.02). The most common co-morbidity for both groups was hypertension, followed by diabetes mellitus and coronary artery disease. There were no significant differences in comorbidities or treatment regimens between the two groups.

**Table 1:** Demographic and clinical characteristics of patients with rhabdomyolysis and patients without rhabdomyolysis * P-value by independent sample t-test for continuous variables and chi-square test for categorical (Fisher’s exact test when expected values are less than 5). N, number; IQR, interquartile range; BMI, body mass index; COPD, chronic obstructive pulmonary disease; DM, diabetes mellitus; ESRD, end-stage renal disease; HTN, hypertension; CAD, coronary artery disease.

	No Rhabdomyolysis (N = 218)	Rhabdomyolysis (N = 22)	P-value^*^
Age median (IQR)	64.8 (55–76)	62 (47–75)	0.21
Sex – N (%)			0.02
Male	125 (57.3)	18 (81.8)	
Female	93 (42.7)	4 (18.2)	
Ethnicities – N (%)			0.10
White	130 (59.6)	8 (36.4)	
African American	39 (17.9)	5 (22.7)	
Hispanic	13 (6.0)	1 (4.5)	
Asian	15 (6.9)	1 (4.5)	
Not reported	21 (9.6)	6 (27.2)	
BMI median (IQR)	28.9 (23.8–32.9)	28.7 (26–32)	0.54
Comorbidities – N (%)			
COPD	22 (10.1)	1 (4.5)	0.70
DM	72 (33.0)	6 (27.2)	0.58
ESRD	8 (3.7)	0 (0)	>0.99
HTN	111 (50.9)	13 (59.1)	0.46
CAD	43 (19.7)	3 (13.6)	0.76
Cancer	2 (0.9)	0 (0)	>0.99
Smoking – N (%)	51 (23.4)	7 (31.8)	0.39
Treatment – N (%)			
Corticosteroids	129 (59.2)	14 (63.6)	0.68
Remdesivir	40 (18.3)	1 (4.5)	0.14
Anticoagulation	39 (17.9)	6 (27.3)	0.26

**Figure 1:**
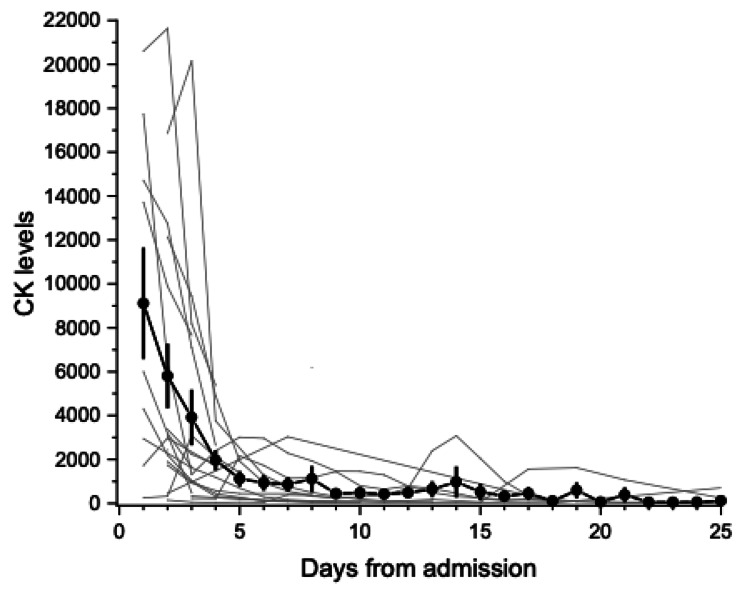
CK levels in rhabdomyolysis COVID-19 patients. Symbols are the mean and SEM for all determinations available on that day. Narrow lines indicate individual patients with linear interpolation of missing points between existing determinations. CK, Creatine kinase; COVID-19, coronavirus disease 2019; SEM, standard error of mean.

Figure 1 shows the trend of CK levels in the rhabdomyolysis group. The majority of patients (86.4%) had their CK levels measured at admission, and almost all patients (95.5%) had their first CK level measurement within the first three days after admission. Median CK levels were 2948 IU/L at admission, 3099 IU/L at peak, and 435 IU/L at discharge. Admission levels were diagnostic of rhabdomyolysis for most patients (72.7%). A median number of days to reach peak levels is the second day of admission with an IQR of 1.25-3 days. Average CK levels were below 1125 U/L by the fifth day of admission, although eight patients had levels that persisted or peaked beyond the fifth day (Figure 1).

Table 2 shows the comparison of outcome between the two groups. There was no statistically significant difference in the in-hospital mortality (23.9% vs 27.3%, p = 0.72), prevalence of severe COVID-19 infection (72.7% vs 54.6%, p = 0.10), presence of AKI (59.1% vs 42.7%, p = 0.14), need for ICU care (72.7% vs 51.4%, p = 0.06), vasopressor support (36.4% vs 25.7%, p = 0.30), and hospital length of stay (19.7 days vs 15.4 days, p = 0.68). However, the need for physical therapy rehabilitation after hospital discharge was more frequently observed in patients who developed rhabdomyolysis (40.9% vs 19.3%, p = 0.02).

**Table 2:** Comparison of outcome between patients with rhabdomyolysis and patients without rhabdomyolysis * P-value by chi-square test for categorical (Fisher’s exact test when expected values are less than 5) and Mann–Whitney test for continuous variables. N, number; COVID-19, coronavirus disease 2019; ICU, intensive care unit.

Outcome	No Rhabdomyolysis (N = 218)	Rhabdomyolysis (N = 22)	P-value^*^
Mortality – N (%)	52 (23.9)	6 (27.3)	0.72
Severe COVID – N (%)	119 (54.6)	16 (72.7)	0.10
ICU stay – N (%)	112 (51.4)	16 (72.7)	0.06
Rehabilitation – N (%)	42/164 (25.6)	9/16 (56.3)	0.02
Acute kidney injury – N (%)	93 (42.7)	13 (59.1)	0.14
Dialysis requirement – N (%)	11 (5.0)	2 (9.1)	0.33
Mechanical ventilation – N (%)	71 (32.6)	13 (59.1)	0.02
Respiratory support – N (%)	180 (82.6)	18 (81.8)	>0.99
Vasopressor use – N (%)	56 (25.7)	8 (36.4)	0.30
Hospital LOS median (IQR) Days	12 (6–21)	12.5 (5–28)	0.68

## Discussion

Our analysis shows that the incidence of rhabdomyolysis was 9.2% in hospitalized patients with COVID-19. Rhabdomyolysis was not associated with increased severity of disease or worse outcomes. However, a significantly higher number of patients had functional limitations in the rhabdomyolysis group as compared to those without rhabdomyolysis.

There is a paucity of data examining the incidence of rhabdomyolysis in patients with COVID-19 infection. The majority of literature is in the form of single case reports. There is only one retrospective study to date examining the incidence and outcome of rhabdomyolysis in hospitalized patients with COVID-19 infection [13]. The reported incidence in this study was nearly two-fold higher (17%) than in the current study (9.6%). The higher incidence of rhabdomyolysis may be due to the difference in the definition used in these two studies. The former study used a cutoff of 1000 IU/L CK levels, whereas we used five times the upper limit of normal (1125 U/L). Our definition is consistent with the previous definition used in the literature [16]. Additionally, there was a significant difference in the demographic characteristics between the two studies. This may also explain the lower incidence of rhabdomyolysis in the current study. Our cohort had relatively younger patients, predominately Caucasian, whereas most patients from the former study were either Black or Hispanic. Moreover, CK levels were not checked in most hospitalized patients, which limits the accuracy of incidence in both studies.

Contrary to the previously published literature, our analysis revealed that rhabdomyolysis is not associated with increased severity of disease or worse outcomes [13]. The difference in the results may be related to the difference in the demographic characteristics and smaller sample size. Both studies did not reveal statistically increased mortality, the requirement for intensive care, or the length of hospital stay in rhabdomyolysis patients. Based on these findings, rhabdomyolysis does not seem to be a predictor of severe COVID-19 infection or worse outcomes.

The degree of CK elevation at admission has been demonstrated to be a predictor for the development of AKI and mortality in rhabdomyolysis before the emergence of the new COVID-19 virus [17]. McMahon’s score is used to predict mortality and development of AKI in patients with rhabdomyolysis [18]. This tool was found to be a predictor for new-onset renal replacement therapy in COVID-19 patients with rhabdomyolysis [13]. COVID-19 infection can potentially cause AKI by either acute tubular necrosis from sepsis or rhabdomyolysis. Approximately 60% of patients with rhabdomyolysis developed AKI during the hospital stay; however, there was no significant difference noted in the prevalence of AKI between the groups due to the small sample size.

The CK level trends revealed in the study demonstrate the early development of rhabdomyolysis. Most patients had elevated levels at presentation, and the levels for almost all patients peaked within three days from admission. This pattern of CK level rise and drop is similar to that demonstrated in rhabdomyolysis from other etiologies [17].

Our study had several limitations. First, this was a small and single-center study. Results may not be generalizable, and larger studies are required to confirm or refute our conclusions. Second, we were not able to ascertain whether rhabdomyolysis was the result of COVID-19, critical illness, or other factors such as the use of steroids or statins. Third, there was inconsistency in measuring CK levels. This potentially could have resulted in an underestimation of rhabdomyolysis incidence as many patients did not have CK levels measured. The opposite may also be true and it is possible that severe illness resulted in more intensive testing, including CK level measurement, causing overestimation of incidence.

## Conclusions

Rhabdomyolysis is a common finding in hospitalized patients with COVID-19 infection, with an incidence of 9.2% based on our findings. Patients with rhabdomyolysis more often require physical rehabilitation at the time of discharge. It is likely that rhabdomyolysis is also associated with more severe disease and a generally worse outcome, although this was not appreciated in our study, likely due to the small sample size. Larger studies with demographically diverse populations are required to determine whether rhabdomyolysis has a significant effect on mortality and severity of COVID-19 infection. If such studies do elucidate such effect, CK levels may potentially be used as a marker to predict severe disease as these levels tend to rise and peak within three days after the presentation of the COVID-19.
